# Opening Pandora’s Box: Exploring Body Image Perceptions and Influencing Factors in Women—An Interpretative Phenomenological Analysis

**DOI:** 10.3390/healthcare13010015

**Published:** 2024-12-24

**Authors:** Konstantina Adamidou, Panagiota Tragantzopoulou

**Affiliations:** 1 Mediterranean College, University of Derby, 54625 Thessaloniki, Greece; k.adamidou@mc-class.gr; 2 School of Social Sciences, University of Westminster, London W1B 2HW, UK

**Keywords:** body image, women, ideal body, cosmetic surgery, qualitative research

## Abstract

**Background/Objectives**: Body dissatisfaction among women has been on the rise, prompting an urgent need to understand the underlying factors influencing their body image. This study explores the perceptions and influencing factors of body image among women in Greek society. **Methods**: Six in-depth interviews were conducted and analyzed using Interpretative Phenomenological Analysis. **Results**: The findings reveal that negative comments from parents and romantic partners, pregnancy, and peer influences are key factors affecting women’s perceptions about their body. Body dissatisfaction was primarily driven by social media and a desire to feel attractive to the opposite gender, leading to extreme behaviors such as excessive exercise, low-calorie intake, and surgical procedures. The aspiration to undergo cosmetic surgeries was significantly influenced by social media portrayals of beauty. On the one hand, the ideal physique for women was characterized by curves and toned muscles, but on the other hand, women described the ideal self as being reconciled with their bodies and less self-critical. **Conclusions**: This study highlights the complex interplay of personal relationships and societal pressures in shaping women’s body image and underscores the need for more qualitative research in this area. Understanding these dynamics is crucial for developing interventions to mitigate the negative physical and mental health repercussions associated with body dissatisfaction.

## 1. Introduction

Many individuals harbor concerns about specific aspects of their bodies, but when these concerns manifest as negative cognitive evaluations, they reflect a negative body image [1]. Body image is understood as a multidimensional construct, incorporating behavioral aspects (such as body-checking behaviors), perceptual aspects (such as the estimation of body size or weight), and cognitive–affective aspects (including attitudes, beliefs, and emotions about one’s body) [1,2]. Negative attitudes and feelings towards one’s body, known as body dissatisfaction, are considered a primary indicator of body-related stress [2,3]. Studies indicate that female adolescents as well as women and men of all ages frequently report body dissatisfaction [4,5]. Nonetheless, research presents mixed findings on body dissatisfaction and its progression with age. Some studies suggest that body dissatisfaction varies across different age groups [6,7], while others indicate that these attitudes remain stable throughout the adult female lifespan [8,9]. Additionally, although body dissatisfaction may stay constant with age in women, the importance placed on appearance tends to diminish over time. For instance, Tiggemann and Lynch [10] discovered that older women placed less emphasis on appearance compared to younger women.

Frederick and colleagues [11] estimated that 20% to 40% of women are dissatisfied with their bodies. Among women, the aspiration for thinness is so common that it has been termed a “normative discontent”, with dieting being a common consequence of body dissatisfaction [12]. Socio-cultural models are widely considered the most robust theoretical frameworks for understanding the emergence of body dissatisfaction and dieting behavior in women and adolescent girls [2,13]. These models suggest that contemporary societal beauty standards emphasize thinness as an ideal, which many women internalize despite its unattainability for most [2]. This thin ideal is propagated and reinforced through various socio-cultural channels, notably parents, peers, and the media.

### 1.1. Influencing Factors and Consequences of Body Image Concerns

#### 1.1.1. Parents, Peers, and Romantic Partners

Parents play a crucial role in shaping their children’s body image [14]. This influence can be direct, through explicit comments about weight or body shape, or indirect, via behaviors such as modeling dieting habits or making remarks that imply dissatisfaction with the child’s eating behavior. The impact of these restrictive or critical messages from caregivers in early life often extends into young adulthood. Recollections of such messages in young adults have been linked to negative body image outcomes, including higher levels of body dissatisfaction, internalized and externalized body shame, and perceived pressure from family to be lean, and disordered eating [15,16,17]. In a qualitative study with 49 individuals, negative weight comments made by their parents during childhood were described as having affected their self-esteem as adults [18].

Given the time spent with peers during adolescence, it is unsurprising that peers play a crucial role in shaping adolescents’ body image perceptions. Adolescents learn about attractiveness by observing and comparing themselves to their peers, gaining insights into which body types are associated with social privilege and popularity [19]. The peer environment allows adolescents to discuss and reinforce appearance-related issues, share behaviors, and criticize those who do not meet certain standards [20]. Research has identified specific mechanisms of peer influence on body image. For instance, body comparisons with peers mediate the relationship between thinness norms and body image concerns in adolescent girls [20]. Peer teasing is also a significant risk factor for body dissatisfaction in both boys and girls over a three-year period [21]. In a qualitative study, participants revealed significant pressure to conform to strict appearance expectations, which were intensified by peers’ surveillance and focus on negative attributes [22]. Conversations about appearance were linked to negative body image perceptions and increased pressure to meet specific ideals. Adolescents also conformed to these ideals to impress opposite-sex peers, valuing themselves based on physical appearance and perceiving themselves as objects for gratification. This finding aligns with the objectification theory [23] which posits that individuals—especially women—tend to view themselves through the lens of others as objects that are evaluated primarily based on physical appearance. However, this study only included Irish individuals, and the focus group method of data collection could have allowed a few dominant individuals to overshadow more withdrawn participants at times.

During adolescence and young adulthood, romantic partners play an additional role in shaping one’s self-perception, including body image [24]. Research indicates that feedback from romantic partners can significantly impact body image. For instance, Goldsmith and Byers [25] found that negative comments from romantic partners decreased confidence and sexual empowerment, which can have profound effects on body image. Similarly, Sheets and Ajmere [26] reported that, within a three-month period, 14% of women and 24% of men were told by their romantic partners to gain or lose weight. Such feedback, whether through direct comments or actions like cuddling, kissing, or pushing away, was perceived as reflective of a partner’s views on one’s body. Despite the well-established link between negative feedback from romantic partners and body image dissatisfaction, qualitative research exploring these experiences remains limited, overlooking this significant factor. Qualitative research could capture the subjective experiences and emotional responses of individuals who receive such feedback. For instance, it could reveal how individuals interpret their partner’s comments or actions, how this feedback interacts with their existing self-perceptions, and how it influences their body image and behaviors over time. Such insights would provide a deeper understanding than is currently available.

#### 1.1.2. Social Media and Beauty Standards

Socio-cultural theories on body image propose that media consumption can lead to body dissatisfaction via two main routes: the internalization of societal beauty standards and the propensity to compare one’s appearance with that of others [2,27]. Internalization involves the degree to which individuals adopt societal beauty ideals as their own personal beliefs and objectives [28]. This field of study posits that, while most individuals are aware of societal beauty standards, not everyone internalizes them to the same extent. Those who do internalize these standards are at a higher risk of experiencing body dissatisfaction and developing eating disorders [13]. The second route involves the social comparison theory, which suggests that people naturally compare themselves to others to assess their progress and standing in various life aspects, including physical attractiveness [29]. These comparisons can be upward (comparing with those perceived as better off) or downward (comparing with those perceived as worse off). Upward comparisons can result in negative feelings, such as increased body dissatisfaction, while downward comparisons can lead to positive feelings, such as reduced body dissatisfaction [30]. However, research indicates that a general tendency to compare one’s appearance with others, regardless of the direction of comparison, can also lead to negative outcomes [31,32].

Social media use has been strongly linked to body dissatisfaction, particularly among young women, as platforms like Instagram and Facebook frequently feature idealized and edited images that reinforce unattainable beauty standards [31]. Instagram, with its emphasis on photo sharing and enhancement tools, uniquely fosters an environment conducive to social comparisons, which can exacerbate body image concerns [33]. The prevalence of filtered and manipulated photos has been shown to contribute to self-objectification and increased dissatisfaction with one’s appearance, underscoring the platform’s potentially harmful effects on mental health. While social media platforms allow users to actively select the content they engage with, such as choosing which accounts to follow [34], users are often influenced by algorithmically generated content that biases their exposure to certain posts [35]. This algorithmic personalization can undermine efforts by users to curate positive or neutral content, as algorithms may still surface undesirable material, including advertisements or content that blurs the line between user-generated and commercial material [34]. On platforms like TikTok, algorithms often rapidly direct users towards increasingly unmoderated or extreme content, as they prioritize engagement by learning which types of posts retain user attention for longer periods [35]. Research has highlighted the harmful consequences of such algorithmic practices. The publication of internal Meta (formerly Facebook) documents indicating that the company was aware of the detrimental mental health effects of its platforms, particularly Instagram, on teenage users [36,37]. For instance, leaked studies demonstrated that one in three teenage girls reported a worsening of body image issues linked to Instagram use [36]. Despite being aware of these outcomes, social media platforms have continued to implement algorithms that exacerbate such issues, often prioritizing user engagement over well-being [37]. Additionally, the addictive nature of social media for some adolescents [38] and the emotionally triggering nature of algorithm-driven content [39] make it particularly difficult for vulnerable users to disengage from harmful material.

Further research by Kleemans et al. [40] underscores the troubling effects of manipulated Instagram photos on adolescent girls’ body image. Their study revealed that exposure to reshaped and retouched selfies led to immediate decreases in body satisfaction, particularly among girls with a strong tendency towards social comparison. Notably, manipulated photos were often perceived as realistic, with reshaping going largely unnoticed, reinforcing the illusion of authenticity. This finding amplifies concerns about the pervasive normalization of digitally altered images on social media and their potential to skew perceptions of reality. Critically, the impact of manipulated images extends beyond mere dissatisfaction to influence significant behavioral intentions, such as the desire for cosmetic surgery. Walker et al. [41] demonstrated that exposure to cosmetically enhanced social media content heightened the consideration of cosmetic procedures, suggesting that these platforms do not simply reinforce body dissatisfaction but actively shape cosmetic aspirations. Supporting this trend, the American Academy of Facial Plastic and Reconstructive Surgery [42] has observed a marked increase in patients influenced by social media esthetics, with influencers playing a critical role in shaping expectations for both surgical and non-surgical interventions.

While research on the connection between social media engagement and facial appearance concerns remains limited, early studies indicate a positive link. Wang and colleagues [43] discovered that frequent viewing of selfies on social media correlates with higher levels of facial dissatisfaction. Furthermore, an experimental study by Fardouly and Rapee [44] demonstrated that undergraduate women who viewed idealized selfies of makeup-wearing women experienced increased facial dissatisfaction and a stronger desire to alter their own appearance compared to those who viewed selfies of women without makeup. These findings suggest that the culture of curated and enhanced beauty on social media platforms can have profound and potentially harmful effects on individuals’ perceptions of their own physical appearance. Given these implications, it is crucial to conduct qualitative studies to gain a deeper understanding of the ways in which social media influences body image and cosmetic surgery considerations. This approach can provide rich, detailed insights into how people internalize and interpret the beauty standards presented on social media, and how these interpretations influence their self-perception and decision making regarding cosmetic surgery. By looking into participants’ subjective experiences, qualitative research can illuminate complexities that might be overlooked in quantitative studies, thus complementing the existing literature and contributing to a more comprehensive understanding of these phenomena.

Research, however, has indicated that the general use of social media may not strongly contribute to body image disturbance. Engaging in diverse activities or interacting with content unrelated to physical appearance, such as viewing posts unrelated to body comparisons, does not typically lead to body dissatisfaction. For example, Fardouly et al. [31] found that brief social media browsing (10 min on Facebook) did not result in significant changes in body dissatisfaction. Similarly, Mabe, Forney, and Keel [45] observed that Facebook browsing for 20 min was linked to a reduction in weight and shape preoccupation, suggesting that general social media engagement may even have a positive effect on body image. However, it is important to note that, in the current study, general social media use was found to significantly relate to body image disturbance, indicating that the broader nature of social media engagement can still have negative effects under certain circumstances.

### 1.2. Positive Body Image and Body Positivity

Before 2005, most research on body image concentrated on negative aspects, such as dissatisfaction with appearance, particularly regarding weight and body shape. However, influenced by the rise of positive psychology and early concepts of positive body image, the idea of body appreciation emerged. Avalos et al. [46] defined body appreciation as encompassing four key elements: having a positive opinion of the body regardless of appearance, accepting imperfections, caring for the body through healthy behaviors, and resisting unrealistic beauty standards promoted by the media. This framework led to the creation of the Body Appreciation Scale (BAS), which highlighted how positive body image contributes uniquely to well-being beyond merely addressing negative body image. By 2010, research on positive body image gained traction, especially with qualitative studies. For example, Wood-Barcalow et al. [47] identified several characteristics of positive body image through interviews with women and body image experts. These included valuing the body for its functionality, embracing a broad definition of beauty, practicing self-care, and filtering negative societal messages. They also described processes like encouraging others to develop positive body image, managing negative influences, and acknowledging that body image can fluctuate. Around the same time, Frisén and Holmqvist [48,49] conducted studies with Swedish adolescents, who highlighted similar themes. These young people appreciated their bodies’ functionality, emphasized personality over appearance, and resisted media-driven beauty ideals.

Building on these findings, Tylka and Wood-Barcalow [50,51] refined the BAS into the Body Appreciation Scale-2 (BAS-2) and clarified what positive body image entails. They argued that positive body image is a multidimensional concept, distinct from negative body image, and integrates acceptance, adaptive behaviors, broad beauty standards, and resilience to negative influences. It is influenced by both internal and external factors, such as relationships, community, and cultural dynamics, and can be adjusted through interventions. Importantly, positive body image is not about being completely satisfied with appearance, narcissistic, or immune to challenges, nor does it neglect self-care or rely on external validation.

In recent years, body positivity has become a widely recognized movement advocating for greater acceptance of diverse body types. At its core, body positivity promotes the idea that “all bodies are good bodies” and encourages individuals to embrace their physical selves [52,53]. Its origins can be traced back to feminist movements of the 1960s, which challenged societal beauty standards and opposed discrimination based on body size [54]. This foundation was further shaped by activism from fat, Black, and queer communities, who sought to address the lack of representation and value assigned to marginalized bodies in visual media [53]. Initially, body positivity emphasized fostering a positive connection with one’s body as a part of a broader value system [55]. While some activists focused on dismantling structural barriers rooted in capitalism, racism, ableism, ageism, sizeism, and patriarchy, the movement predominantly framed body acceptance as an individual responsibility and political statement, rather than a call to address systemic inequalities such as fat stigma or white supremacy. This individualistic framing led to the appropriation and commercialization of body positivity by mainstream industries, such as fitness and wellness, which often excluded bodies that are fat, non-white, differently abled, or nonheteronormative [53]. In social media spaces, body positivity hashtags (e.g., #allbodiesaregoodbodies or #effyourbeautystandards) aim to celebrate body diversity and encourage self-acceptance and care. However, the movement’s shift away from its activist roots has created a significant limitation: its messaging frequently prioritizes and showcases bodies that align with societal beauty standards, such as those that are young, white, thin, able-bodied, or cisgender, thereby marginalizing the very groups it originally sought to empower [56].

The rise in the number of women reporting body dissatisfaction and resorting to cosmetic surgeries underscores the growing pressure women face to conform to narrow and often unattainable societal beauty standards. These pressures are further magnified by globalized ideals of appearance, perpetuated by media and social platforms, which often marginalize diverse body types. Despite the prevalence of these issues, there is a notable lack of qualitative studies that deeply explore the personal experiences and perceptions of women regarding their body image, particularly within the cultural context of Greek society, where traditional values and modern influences intersect. In recent years, the body positivity movement has emerged as a powerful counter-narrative to societal pressures, emphasizing the acceptance of all body types and challenging rigid beauty ideals. While this movement has sparked important conversations and gained significant visibility, it remains unclear how its principles resonate within specific cultural contexts like Greece. Given that the body positivity movement has largely centered on Western discourses, its impact on women’s perceptions of body image in other cultural settings warrants closer examination. This study, therefore, seeks to fill a critical gap in the literature by exploring the perceptions of body image among Greek women and identifying the factors that shape these perceptions. Understanding these dynamics is essential for developing culturally sensitive interventions and support mechanisms that promote healthier body image and well-being among women. The study aims to address the following questions: (1) what are the perceptions of body image among Greek women of different ages? and (2) what factors shape how women think about and feel regarding their bodies?

## 2. Materials and Methods

### 2.1. Design

The study design, data collection, and analysis were guided by Interpretative Phenomenological Analysis (IPA). A crucial aspect of IPA is its “double hermeneutic” nature, where a researcher interprets each participant’s recounting and understanding of their own experiences [57].

### 2.2. Participants

Following ethical approval by the ethics committee of the authors’ institution, recruitment was initiated. Participants were recruited via personal invitation and expressed interest in participating after a relevant advertising poster was uploaded on the primary researcher’s social media accounts. The personal invitations were extended to individuals known to meet the inclusion criteria—Greek women above the age of 18—such as acquaintances or contacts within the researcher’s network who were then asked to share the advertisement with others. This snowballing approach helped to identify potential participants who expressed interest and met the eligibility criteria. To ensure a homogenous sample, we followed the idiographic principle of IPA, which requires small, carefully selected samples for in-depth, case-by-case analysis. In line with Smith et al.’s [57] guidance on achieving homogeneity, we sought participants with similar demographic and socioeconomic profiles, focusing on a specific small city in Greece. While the inclusion criteria were broad in terms of age, we also aimed to achieve representation across different age groups by recruiting women from each of three age brackets (e.g., 20s, 30s, and 40s). The recruitment process resulted in a fairly balanced distribution across these age groups, with two participants from each bracket. This natural outcome helped ensure representation while maintaining a homogenous sample. Exclusion criteria ensured that participants did not have a diagnosed eating disorder or other mental health issues, as these conditions could significantly influence their experiences and would not align with our aim to focus on broader societal and cultural factors affecting eating behaviors. As a result, our sample comprised six women who identified as heterosexual, white, cisgender, and were fairly comparable in terms of demographic/socioeconomic status (Table 1). This alignment supported our goal of conducting detailed, idiographic analysis consistent with IPA guidelines [58].

### 2.3. Data Collection

All participants gave written consent to take part in the study and participated in semi-structured interviews led by the primary researcher. Depending on each participant’s preference and availability, interviews were held either in person or via Teams and were fully audio-recorded. The interview guide was created following IPA guidelines [58], informed by themes from the existing literature, and in consultation with the second researcher. The interview questions were designed to be broad and open-ended to explore participants’ perceptions of their body image, how these perceptions might have evolved over time, any experiences that influenced their body image, and their description of an ideal body. Participants were encouraged to share their experiences in detail, with prompts used when necessary to guide them from general descriptions to specific and emotional reflections. Each interview began with an unrecorded informal discussion to build rapport, reduce anxiety, outline the interview plan, and confirm consent to record. Interviews only started after obtaining oral consent and they lasted between 30 and 90 min. At the end of each session, the researcher reminded participants of the study’s aims, provided an opportunity for questions, and expressed gratitude for their involvement.

### 2.4. Data Analysis

To explore the body image perceptions of women and gain insight into our participants’ experiences, we utilized IPA [57]. This approach emphasizes understanding individuals’ perceptions and narratives about objects and events rather than categorizing phenomena based on predefined systems or scientific criteria [59]. Interviews were transcribed verbatim and anonymized by the first author. Then, they were analyzed manually following the procedures of IPA as detailed by Smith et al. [58]. The analysis began with multiple readings of the first transcript, during which exploratory notes were taken to gain a comprehensive understanding of the participant’s account. This open textual analysis focused on identifying significant statements, language use, distinctive phrases, emotional responses, and the context of their experiences. From these notes, higher-level conceptual themes were identified. These themes were then grouped based on conceptual similarities and further refined to define their interrelationships. To ensure rigor, these initial themes were organized into a coding tree, categorizing them into groups based on conceptual similarities and hierarchical relationships. For example, initial codes such as “pressure to conform” and “cosmetic surgery desire” were grouped into a broader theme labeled “appearance-related pressures”. The development of these thematic groupings was iterative, requiring repeated engagement with the transcripts to refine and verify their coherence with the raw data.

The first author developed the initial notes and themes, which were reviewed by the second author to ensure qualitative rigor and coherence. This process was repeated for each participant to ensure that their lived experiences were fully captured. Finally, the identified themes from each of the six cases were clustered based on their similarities and interrelationships. The second author further refined these groupings to create a master table of superordinate themes that best represented the participants’ experiences. Disagreements between researchers regarding the coding or thematic grouping were resolved through collaborative discussions. To enhance the validity of the findings, member checking was employed. Participants were provided with summaries of the identified themes and asked for feedback on whether these accurately reflected their experiences. Their feedback informed the final refinement of the themes and ensured that interpretations remained grounded in participants’ narratives. Quotations from the interviews were used to support the findings and analysis.

In IPA, researchers play a critical role in interpreting the phenomenon [58], making reflexivity crucial. Reflexivity involves researchers critically examining and being aware of their own biases and perspectives throughout the research process [60]. The researchers’ interest in women’s body image, along with their personal experiences of being women and their relationship with eating and body image, also influenced their perspective. These pre-existing understandings could both enrich and limit the research process, such as influencing decisions to probe further into certain experiences during interviews or shaping the interpretation of data during analysis. To maintain reflexivity, both researchers kept a reflexive diary throughout the study, as recommended by Newton et al. [61]. For example, one researcher noted feeling a strong resonance with a participant’s narrative about dieting pressures, which prompted further scrutiny of how this personal identification might influence coding decisions. Reflexive discussions between researchers, alongside the diaries, ensured ongoing critical reflection and minimized the impact of personal biases on data interpretation.

## 3. Results

In total, three superordinate themes were identified, and each theme had distinct subordinate themes that provided insights on the research questions (see Table 2).

### 3.1. Factors Affecting Body Image

This theme explores how interpersonal relationships and life as well as physical changes during pregnancy shape and impact individuals’ perceptions of their bodies.

#### 3.1.1. Toxic Romantic Relationships

Five participants described a past romantic partner who would negatively comment on their body, their appearance, or their eating habits. Nicole felt that her partner consistently targeted her insecurities, intentionally hurting her in areas where she was most vulnerable. She recounted how he would compare her unfavorably to others, criticizing her body by saying things like “*you don’t have a butt, she does*” and urging her to go to the gym to do squats to build one. Reflecting on this experience, she explained the impact her partner had on her self-perception, not only regarding her body but also her face:

“*This particular person greatly influenced how I see myself, not only in terms of my body but also my face. He started saying things like, “put on some makeup,” because I wasn’t someone who used makeup very much.*”

The powerful impact that romantic relationships can have on an individual’s body image was also acknowledged by Olivia. Her accounts describe a partner who constantly bombarded her with guilt, criticizing her choices and making her feel ashamed for eating or not taking care of herself according to his standards. She recalls being told “*you can’t wear that*” or “*what you’re wearing doesn’t look good, we can’t go out like that*”, which felt like a deep sense of control and disapproval from her partner.

Some participants tried to understand the reasons behind their partners’ negative comments, perceiving them as attempts to undermine their confidence. Antigoni was certain that her past partner’s intention was to lower her confidence by calling her “*fat*” even though she was thin. Athina elaborated on this, suggesting that the aim was to keep her confined within the relationship by making her believe that no other man would be interested in her, thus convincing her that her partner was the best option she could have. She described the relationship as “*very toxic*”, highlighting how her boyfriend’s behavior influenced her self-worth and confidence. By making her believe that she could only have him by her side due to her appearance, Athina thought that her boyfriend affected her self-esteem and perpetuated a negative body image:

“*It [the relationship] was very toxic…making me believe that this is who I am and that I could only have him as a person by my side, in a relationship. He obviously didn’t love who I was, at least externally, and he tried to tell me that…that I wasn’t good enough to be by him side, but that I could only have him given how I looked.*”

#### 3.1.2. Significant Others and Pregnancy

A few participants recounted comments from their family, particularly from their mothers, about their bodies. Nicole stated that her mother would “*make a comment, Oh, you are eating crisps again, leave it, you’ll be this and that*”. Irene also described comments from her mother who instilled a strong belief that women must be very thin. This belief was communicated explicitly and frequently, especially when Irene gained weight:

“*My mother grew up with a very strong idea that women must be very thin. And many times, when I gained weight, my mother would mention it to me, saying things like, Oh my goodness, look at your arms, why have they become like this? You need to do something about it.*”

For half of the women, these familial comments created a sense of pressure to conform to an idealized body image. The impact of these maternal beliefs was profound, often leading participants to feel a need to “meet certain standards of beauty”, not only for their own self-esteem but also to avoid negative judgments from close family members. This often involved an internalized sense of body dissatisfaction and a desire to meet specific physical ideals.

Friends were thought to be an additional factor affecting body image. Nicole describes discussions with friends about exercise and weight loss, thinking that “*my friends are stricter, have clear goals. I would like to be a little thinner or tighter and these discussions might get me in the mood to lose weight or exercise*”. This reflects how peer pressure can create a competitive environment where body image is scrutinized, and women feel motivated to meet certain physical expectations in order to align with the group or be accepted. Similarly, Olivia’s experience highlights how peer comments on appearance can have a lasting effect on body image. She recalled the following:

“*The other thing I remember is that my friends in high school, because I had several boyfriends, never saw me as the girl they liked. And many times, they would comment, ’You have neither tits nor ass.*”

In this narrative, the emphasis on lacking certain physical attributes, particularly when comparing herself to other girls, was described as contributing to Olivia’s feelings of inadequacy. This type of peer commentary was thought to significantly affect body image by reinforcing the idea that physical appearance is central to one’s worth and attractiveness. The recollection of these hurtful remarks was viewed by Olivia as leading her to doubt her attractiveness and to reduce her self-confidence regarding her body.

Finally, two participants mentioned the inevitable changes in their bodies during pregnancy. Irene expressed the difficulty of seeing weight gain every month, while Kate expressed dissatisfaction with her post-pregnancy body. Observing her skin’s relaxed state, Kate felt disappointed and strongly desired to return to her pre-pregnancy appearance. Nicole also expressed dissatisfaction with her current body image, stating it does not align with her pre-pregnancy image. Consequently, she was actively seeking to improve and rectify it through exercise, diet, and skincare products, primarily focusing on regaining her pre-pregnancy appearance:

“*It troubles me a bit because after pregnancy my body has relaxed quite a bit. And I don’t have the image I had before, so I’m trying to fix and correct that. Mainly with exercise and diet. And I also use some body cosmetics, mainly for the skin because, um, during pregnancy, I experienced a big shock, so I need to, I need to fix it in any way I can.*”

### 3.2. Struggling to Fit

This theme encapsulates the struggles participants faced as they tried to align with societal expectations of beauty, dealing with desires for acceptance, the pursuit of esthetic enhancements, and their conceptions of the ideal physique.

#### 3.2.1. Wanting to Be Wanted

The longing for acceptance and desirability was a common thread among all six women, leading to the emergence of negative emotions and thoughts. This desire was described as being deeply ingrained in their daily experiences, where the emphasis on appearance dominated their perception of the world. Olivia considered the pursuit of attractiveness a “*natural inclination*”. Maintaining a favorable image for others was not only desired but also perceived as an unspoken standard in the lives of women. Antigoni’s desire to feel wanted was evident in her daily interactions, whether at work or with her partner, and especially in situations where exposure and visibility are key. She found it “*challenging to feel comfortable and desired*” particularly in group settings or with her partner, where her body image was viewed as playing a significant role in seeking acceptance. Nicole also described dealing with feelings of insecurity and melancholy when her body size seemingly affected her desirability. She consistently entertained intrusive thoughts about whether being thinner would garner more attention from men, particularly when witnessing a thinner friend receiving such attention. This discrepancy in attention felt like a blow to her desire for acceptance and attraction, with her weight acting as a perceived barrier to achieving it:

“*I may think sometimes if I am out with friends and someone happens to come and talk to a friend, I may think, ’Ah, she is thinner, she is more beautiful’. It’s an insecurity that I feel now…that if I were a little bit thinner, guys might talk to me as well.*”

#### 3.2.2. Pursuit of Esthetic Surgery

Many participants expressed a strong desire to change their bodies, including areas like their arms, breast, bottom, or face, such as their nose and teeth, through plastic surgeries, or admitted to having done so. For example, Irene shared that she changed her nose, a decision she never regretted as it was something she “*really, really wanted to do*”. Antigoni emphasized the impact of appearance on self-esteem, particularly mentioning her rhinoplasty, which she believed made her more attractive, boosted her confidence, and helped her love herself more. Nicole shared that she underwent multiple dental procedures to enhance her smile, viewing it as crucial for her appearance and self-presentation, especially on social media. The following quote reveals an ongoing struggle with self-image and the pressure to meet specific standards, leading to multiple cosmetic interventions:

“*So, I have gone through the process of altering them twice, and now I am going through it for a third time, because I consider it an important aspect of one’s appearance. It has to do with your smile and how you present yourself in photos and, in general, on social media.*”

Similarly, Kate shared her internal struggle with wanting to change her body, specifically her chest and arms, to feel more comfortable, highlighting her ongoing goal to undergo arm surgery to feel better in sleeveless tops:

“*I want to fix my arms…my desire is to have plastic surgery on my arms. Just for myself, to feel better, because I want to wear sleeveless shirts. Because I want to see my arms once the way I want to see them. I would like to fix that. And it’s my goal that I will fix it. I’ve set it as a goal.*”

#### 3.2.3. The Desired Physique

Participants expressed diverse perceptions of the desired physique, fluctuating between notions of fitness and curvaceousness. Some emphasized the desire for a fitter physique, characterized by visible muscle tone, indicating a commitment to exercise and physical well-being. For instance, Irene expressed, “*I would like to be a little bit stronger…fitter. To have a body where the muscles are more visible, one that appears to be actively exercising*”. Athina described the confidence she felt when her muscles were visible. Conversely, some participants desired curves, either in the buttocks or the chest. Nicole described her body as average because she lacks “*large buttocks or breasts, unlike many other women*”. Similarly, Antigoni envisions the ideal body as curvy, akin to those portrayed on social media. Despite acknowledging the prevalence of highly edited or surgically enhanced bodies online, Antigoni admits to feeling inadequate and unattractive in comparison:

“*I don’t feel beautiful seeing my body like that. Also, because many of us now have social media, and you see many women who might be using filters, might have had surgeries, might be anything, projecting a very curvy body, a very, how should I put it, um, feminine body, which I cannot have…seeing this makes me feel that I am not attractive enough to attract men.*”

### 3.3. Making Peace with the Mirror

This theme reflects the participants’ ongoing journey towards reconciling with their body image, moving from strict self-criticism in their younger years to a more holistic and accepting view of themselves as they strive for a more peaceful and balanced self-concept.

#### 3.3.1. The Younger, the Stricter

Several participants recounted being stringent regarding their bodies and selves during their youth. Kate expressed feeling “*vulnerable*” about her body during her younger years, which prompted her to adopt strict self-discipline, mistakenly perceiving herself as overweight despite being slender. Nicole shared a similar experience, recalling how she believed she had “*fat legs*” during her youth, despite weighing only 52 kg. Antigoni reflected on her past perceptions of her body image and how they have evolved over time. She acknowledged being strict about her appearance during her adolescence, and described her previous self-judgment as less caring and more critical, indicating a lack of self-compassion. Looking back at old photographs, she realized that her younger self perceived herself as larger than she actually was. However, she now adopts a more realistic and accepting perspective towards her body, acknowledging that her current image aligns better with reality:

“*I think when I was 13–15, I was a bit stricter. I remember taking pictures in general and thinking that I was larger than I am. Now, when I look back at those photos, I realize I was very thin then. So, now my perception is more objective... it’s closer to the reality of the image I have for my body compared to when I was younger.*”

In their youth, two participants’ strict approach to body management led them to extreme measures. Irene recounted a phase of intense exercise commitment, spending extensive hours at the gym, to the detriment of her well-being, even experiencing a cessation of her menstrual cycle. Seeking guidance, she consulted a doctor to rectify the situation. Similarly, Nicole endured the cessation of her period due to extreme and exhausting dieting practices. Nicole also experienced unexpected weight loss and alterations in her body shape, reflecting a lack of awareness regarding the harm inflicted on her body by these extreme measures:

“*I followed some completely foolish and exhausting diets. Like for example, eating a yogurt a day or an apple…The obsessions you have at these young ages, resulting in strange weight loss, like suddenly losing from the chest I had, a very nice and full chest. Also, my period stopped for 7 months during that period of my life, obviously not realizing that I was harming my body.*”

#### 3.3.2. The Ideal Self

The ideal self was described as embodying self-acceptance and a holistic way of valuing, rather than evaluating, oneself. For Nicole, the ideal self is characterized by achieving total self-acceptance regarding her appearance. Although she acknowledged that she has not yet reached this final stage of personal development, she identified it as the ultimate goal she aspires to achieve. Her ideal self was conceptualized as one where she recognizes her uniqueness, attractiveness, and intrinsic value, embracing herself exactly as she was born. This inner peace was described as arising from accepting her complete appearance and feeling content and happy with it. The ideal self, therefore, represents a state of mind where she takes pride in who she is, rather than desiring any changes:

“*I think the way I am…I’m perfect for me. Namely, there is no ideal for any human being. I was born this way, this is how I should be, I should feel enough just the way I am for me. So, I don’t think the ideal would be to change something about myself. I think the ideal is to put in my mind that the way I am is totally enough.*”

Athina experienced the ideal self as a reconciliation with both time and her body. She emphasized the importance of self-care through a healthy lifestyle, including nutritious meals, exercise, and skincare, practiced without exaggeration. The ideal self was also experienced as a holistic approach that includes education, good mental health, and being at peace with oneself. Kate, for instance, prioritized these factors, placing physical appearance secondary. She described true strength as stemming from self-cultivation and general self-improvement. For her, the ideal self was a comprehensive concept, where body image is no longer the central focus but rather a space in which she can live peacefully:

“*I think it must be a combination of things… the most important thing is education and mental health… if you have these two, and you are strong enough anyway, then you can achieve things in your life, and you have such will and appetite that everything else will follow.*”

## 4. Discussion

The present study explored body image perceptions among women, focusing on their experiences and influencing factors. The analysis identified negative comments from parents and romantic partners, peer influences, and pregnancy as key factors across all age groups affecting body image, consistent with the existing literature [15,20,25]. Among these factors, younger participants most frequently referred to romantic partners, noting that their negative comments led to low self-esteem, guilt about eating, and body shame. A novel finding of our study was the perceived motive behind these comments. Participants believed their partners aimed to confine them in the relationship by making them feel unattractive to other men. The negative comments about their bodies were described as making the women feel less confident and diminishing their self-worth. Interestingly, no participant referred to positive comments made by their partners, which could suggest that negative comments may have a more lasting effect. This absence of positive reinforcement in the relationships could reflect how damaging and impactful negative remarks are to women’s self-perception and body image. Demeaning comments and body shaming can be considered forms of emotional abuse [56], significantly impacting women’s body image throughout their lives, particularly when they come from romantic partners. Body shaming by romantic partners may lead to unhealthy practices by women who might strive to receive the acceptance and admiration of their partners. However, as a qualitative study, our findings suggest that the motives behind body shaming are likely more complex and multifaceted. While control and confinement emerged as dominant themes, participants also described their partners’ actions in ways that hinted at insecurities and societal pressures. This could suggest that some comments might stem from internalized societal beauty standards or the partner’s own struggles with self-image. Critically, this highlights the need to approach body shaming not only as a relational dynamic but also as a part of a broader cultural context where body image and value are heavily commodified.

All participants in this study expressed a strong longing to feel desired by men, often leading to feelings of disappointment and melancholy when they felt their bodies did not meet the beauty standards expected by men. Consequently, some participants shared a desire to lose more weight or change their appearance. These findings illustrate the deep-rooted self-objectification in women. From a very young age, women learn to view and value their bodies as objects that will garner appreciation from the opposite gender, prioritizing their physical appearance over other personal characteristics [23,62,63]. This aligns with findings from Holland and Tiggemann [64], which highlight how thin-ideal media exposure perpetuates the societal pressure on women to conform to unrealistic beauty standards, contributing to body dissatisfaction and maladaptive behaviors. As societies continue to objectify women and the media sexualizes them, women are perceived to have less agency and are viewed as less deserving of moral treatment [65]. This societal pressure is not limited to Western contexts, as globalized media continues to export and reinforce these ideals across cultures. Holland and Tiggemann [64] emphasize the pervasive influence of media in shaping and spreading beauty standards that prioritize thinness and sexualization, leading to the internalization of these ideals among women worldwide. This societal pressure leads women to devalue their own bodies and seek changes to feel accepted in a world that values adherence to Western beauty standards [64]. This intense pressure may contribute to body dissatisfaction, extreme eating practices, and behaviors such as addiction to cosmetic surgeries, affecting women’s mental and physical well-being.

Our research findings indicate that the desire to undergo cosmetic surgery is prominent across all age groups, and is driven by social media and body image concerns. These findings support previous research suggesting that the ability to compare oneself with edited photos and people on social media increases body and facial dissatisfaction, prompting individuals to pursue esthetic procedures [42,44]. Furthermore, the number of women who either wish to undergo cosmetic surgery or have already proceeded with multiple surgeries to achieve these standards has significantly increased [66,67]. Drawing upon the social comparison theory [29], these upward comparisons with women perceived as epitomizing an ideal standard of beauty foster feelings of inadequacy regarding one’s own perceived attractiveness, consequently exacerbating body dissatisfaction. This may lead women to perceive cosmetic surgery as a means to ascend within the beauty hierarchy on social media, perpetuating the illusion that such procedures offer an opportunity for advancement. The popularization of cosmetic surgeries is alarming, as it not only perpetuates unrealistic beauty ideals but also poses serious health risks.

A novel finding of our research was the perceived ideal physique described by women. Unlike the thin ideal commonly cited in the literature [2,20], the women in our study described a curvy and fit body as the body type that they regarded as attractive. This suggests a shift in the perceived ideal female body in this particular sample, reflecting the evolving nature of societal beauty standards. However, given the small and homogeneous nature of our sample, these findings should be viewed with caution and may not fully represent broader cultural trends or other populations. The body ideals women develop depend on the female bodies they are regularly exposed to and the internalization of these ideals [68,69]. Therefore, women today may be exposed to more curvy and fit bodies, shaping their desire to conform to these standards. This trend can also be explained by the increasing promotion of healthy eating and fitness on websites, which encourages individuals to adopt healthy eating habits [70] as well as by the social media culture of fitness that prompts individuals to work towards a fit and athletic body [71]. Fitspiration websites and the evolving fitness culture on social media have further amplified this shift by playing a significant role in shaping modern ideals of health and fitness. These platforms, which aim to motivate individuals to live healthier lifestyles, often blend visually striking images with motivational texts related to exercise and diet [72,73]. However, they also promote specific narratives and ideals that can profoundly impact people’s perceptions of health, fitness, and body image. One prominent narrative is the shift from the traditional thin ideal to the “strong is the new skinny” mantra [72,73]. This concept emphasizes physical strength and muscularity over the extreme thinness once glorified in earlier media. While this might appear to be a positive move away from unhealthy body standards, it can still promote an overemphasis on physical appearance, often intertwining the notions of strength with leanness and esthetic perfection. This creates a new form of pressure, as individuals may feel the need to conform to highly specific, appearance-driven fitness ideals. This culture can reinforce problematic beliefs, such as overvaluing appearance, fostering eating concerns, and normalizing excessive exercise, all of which negatively affect mental health and self-esteem [33,72,73]. While the intention of fitspiration websites is to inspire, they inadvertently contribute to the reinforcement of unrealistic and narrowly defined fitness ideals that may escalate body image dissatisfaction.

Throughout the analysis, participants discussed being stricter with their bodies and themselves during their younger years, particularly during adolescence, in order to achieve their ideal physique. This led them to extreme strategies, such as drastically limiting their calorie intake or engaging in excessive exercise. These extremes were thought to contribute to serious health issues, such as the cessation of menstruation. Engaging in such behaviors can have severe repercussions on women’s health, potentially leading to eating disorders [74,75,76,77]. However, older participants in this study shared that they are no longer as strict with their bodies and do not engage in these extremes anymore. These findings align to some extent with the literature which suggests that older women place less emphasis on appearance [10], contradicting previous research indicating stable body dissatisfaction throughout life [8,9]. Nonetheless, a novel finding of our study was the ideal self described by all participants, particularly emphasized by older women, who experienced it as a more liberating way of thinking about the body. For these women, the ideal self represented a state of mind in which they felt proud of who they were, rather than desiring to change their bodies. This self-acceptance was deeply tied to prioritizing aspects of life beyond appearance, such as health, education, and mental well-being. This finding is partly supported by previous qualitative research which showed that some women felt happier as they aged due to reduced societal pressure to meet specific beauty ideals, a more holistic assessment of their bodies, and a shift in focus towards more important aspects of life beyond appearance [78]. The shift towards a holistic approach to self-valuation, focusing on functionality and self-care rather than conforming to societal beauty standards, aligns closely with the principles of positive body image and the body positivity movement. Body positivity, as discussed in the literature, emerged from feminist and activist roots that challenged societal beauty norms and sought to empower individuals to embrace diverse body types. In line with this, the women in our study emphasized the importance of self-acceptance and resisting external pressures related to appearance. Many participants, particularly those of older ages, described how their views on body image had evolved into a more inclusive and self-compassionate approach, aligning with the idea that “all bodies are good bodies”. This reflects the shift in body image research from a focus on dissatisfaction with appearance to a more positive framework, emphasizing body appreciation, self-care, and resilience against societal pressures [46,47]. Interestingly, the ideal self described by participants in this study was not about achieving a certain physical standard, but about embracing one’s body for its functionality and prioritizing overall well-being. This mirrors findings in body image research that highlight the importance of valuing the body for its capabilities, resisting the unrealistic beauty ideals promoted by the media, and fostering a more inclusive definition of beauty that embraces diversity. While body positivity has become a movement focused on the acceptance of diverse bodies, our participants’ descriptions of their ideal selves suggest that this shift towards self-acceptance and well-being can be seen as a personal journey that transcends age, shaped by individual experiences and ongoing personal growth [47,48,49].

This study has explored women’s perceptions of their body image and the factors that may lead them to want to change their bodies and appearance. The use of IPA methodology assumes that the findings are contextually bound, which inherently limits the cultural transferability of the results. Specifically, the research conclusions are based exclusively on women raised and living within a specific city in Greece, shaped by its unique socio-cultural environment. As such, the findings may not be directly transferable to women from other cities, larger urban centers, or different cultural contexts, where socio-cultural pressures related to body image may vary significantly. Additionally, women above the age of 50 were not included, restricting our understanding of how women in this age group may have different body image perceptions and influences. Future studies should explore this topic in older women and across diverse cultures. Research could investigate the influence of negative feedback from romantic partners on women’s desire for esthetic procedures, both surgical and non-surgical. Importantly, more qualitative studies are needed to capture first-hand experiences of the evolving pressures related to body and appearance that women face.

## 5. Conclusions

In conclusion, this study provides valuable insights into the factors shaping women’s body image perceptions, with a particular focus on the impact of negative feedback from romantic partners, societal beauty standards, and social media. The findings highlight the profound emotional and psychological effects of body shaming, as well as the significant role social media plays in fueling body dissatisfaction and the desire for cosmetic surgery. While body dissatisfaction was described across all age groups, all six women in the study, but more emphatically those in the older groups, expressed a shift towards greater self-acceptance and a more holistic approach to body valuation, emphasizing self-care, health, and personal growth over appearance. These results contribute to a deeper understanding of the complex ways in which women internalize societal pressures and develop their body image, illustrating the need for ongoing attention to the emotional well-being of women as they navigate evolving beauty standards.

## Figures and Tables

**Table 1:** Participant demographic information.

Participants	Pseudonyms	Age Group	Weight Range	Education Level	Marital Status
P1	Antigoni	20–29	70–75 kg	High school degree	Single
P2	Olivia	20–29	55–60 kg	Bachelor’s degree	Single
P3	Nicole	30–39	65–70 kg	Master’s degree	Married
P4	Irene	30–39	90–95 kg	High school degree	Married
P5	Athina	40–49	55–60 kg	College degree	Married
P6	Kate	40–49	85–90 kg	Master’s degree	Divorced

**Table 2:** Superordinate and subordinate themes.

Superordinate Themes	Subordinate Themes
Theme 1: Factors Affecting Body Image	Toxic Romantic Relationships
	Significant Others and Pregnancy
Theme 2: Struggling to Fit	Wanting to Be Wanted
	Pursuit of Esthetic Surgery
	The Desired Physique
Theme 3: Making Peace with the Mirror	The Younger, The Stricter
	The Ideal Self

## Data Availability

Data will be available upon reasonable request.

## References

[1] Cash T.F. (2004). Body image: Past, present, and future. Body Image.

[2] Thompson J.K., Heinberg L., Altabe M., Tantleff-Dunn S. (1999). Exacting Beauty: Theory, Assessment, and Treatment of Body Image Disturbance.

[3] Grogan S. (2022). Body Image: Understanding Body Dissatisfaction in Men, Women and Children.

[4] Duncan M.J., Al-Nakeeb Y., Nevill A.M., Jones M.V. (2006). Body dissatisfaction, body fat, and physical activity in British children. Int. J. Pediatr. Obes..

[5] Mond J., Mitchison D., Latner J., Hay P., Owen C., Rodgers B. (2013). Quality of life impairment associated with body dissatisfaction in a general population sample of women. BMC Public Health.

[6] Baker L., Gringart E. (2009). Body image and self-esteem in older adulthood. Ageing Soc..

[7] Esnaola I., Rodríguez A., Goñi A. (2010). Body dissatisfaction and perceived sociocultural pressures: Gender and age differences. Salud Ment..

[8] Fallon E.A., Harris B.S., Johnson P. (2014). Prevalence of body dissatisfaction among a United States adult sample. Eat. Behav..

[9] Tiggemann M., McCourt A. (2013). Body appreciation in adult women: Relationships with age and body satisfaction. Body Image.

[10] Tiggemann M., Lynch J.E. (2001). Body image across the life span in adult women: The role of self-objectification. Dev. Psychol..

[11] Frederick D.A., Jafary A.M., Gruys K., Daniels E.A. (2012). Surveys and the epidemiology of body image dissatisfaction. Encycl. Body Image Hum. Appear..

[12] Rodin J., Silberstein L., Striegel-Moore R. (1984). Women and weight: A normative discontent. Nebraska Symp. Motiv..

[13] Stice E. (2002). Risk and maintenance factors for eating pathology. Psychol. Bull..

[14] Rodgers R., Chabrol H. (2009). Parental attitudes, body image disturbance, and disordered eating amongst adolescents and young adults: A review. Eur. Eat. Disord. Rev..

[15] Oliveira S., Marta-Simões J., Ferreira C. (2019). Early parental eating messages and disordered eating: The role of body shame and inflexible eating. J. Psychol..

[16] MacDonald D.E., Dimitropoulos G., Royal S., Polanco A., Dionne M.M. (2015). The family fat talk questionnaire: Development and psychometric properties of a measure of fat talk behaviors within the family context. Body Image.

[17] Wansink B., Latimer L.A., Pope L. (2017). “Don’t eat so much”: How parent comments relate to female weight satisfaction. Eat. Weight Disord..

[18] Eli K., Howell K., Fisher P.A., Nowicka P. (2014). “Those comments last forever”: Parents and grandparents of preschoolers recount how they became aware of their own body weights as children. PLoS ONE.

[19] Carey R.N., Donaghue N., Broderick P. (2014). Body image concern among Australian adolescent girls: The role of body comparisons with models and peers. Body Image.

[20] Carey R.N., Donaghue N., Broderick P. (2013). Peer culture and body image concern among Australian adolescent girls: A hierarchical linear modelling analysis. Sex Roles.

[21] Paxton S.J., Eisenberg M.E., Neumark-Sztainer D. (2006). Prospective predictors of body dissatisfaction in adolescent girls and boys: A five-year longitudinal study. Dev. Psychol..

[22] Kenny U., O’Malley-Keighran M.-P., Molcho M., Kelly C. (2017). Peer influences on adolescent body image: Friends or foes?. J. Adolesc. Res..

[23] Fredrickson B.L., Roberts T.A. (1997). Objectification theory. Psychol. Women Q..

[24] Holsen I., Jones D.C., Birkeland M.S. (2012). Body image satisfaction among Norwegian adolescents and young adults: A longitudinal study of the influence of interpersonal relationships and BMI. Body Image.

[25] Goldsmith K.M., Byers E.S. (2016). Perceived impact of body feedback from romantic partners on young adults’ body image and sexual well-being. Body Image.

[26] Sheets V., Ajmere K. (2005). Are romantic partners a source of college students’ weight concern?. Eat. Behav..

[27] van den Berg P., Thompson J.K., Obremski-Brandon K., Coovert M. (2002). The tripartite influence model of body image and eating disturbance: A covariance structure modeling investigation testing the mediational role of appearance comparison. J. Psychosom. Res..

[28] Thompson J.K., Stice E. (2001). Thin-ideal internalization: Mounting evidence for a new risk factor for body-image disturbance and eating pathology. Curr. Dir. Psychol. Sci..

[29] Festinger L. (1954). A theory of social comparison processes. Hum. Relat..

[30] Leahey T.M., Crowther J.H. (2008). An ecological momentary assessment of comparison target as a moderator of the effects of appearance-focused social comparisons. Body Image.

[31] Fardouly J., Diedrichs P.C., Vartanian L.R., Halliwell E. (2015). The mediating role of appearance comparisons in the relationship between media usage and self-objectification in young women. Psychol. Women Q..

[32] Halliwell E., Harvey M. (2006). Examination of a sociocultural model of disordered eating among male and female adolescents. Br. J. Health Psychol..

[33] Tiggemann M., Zaccardo M. (2015). “Exercise to be fit, not skinny”: The effect of fitspiration imagery on women’s body image. Body Image.

[34] Perloff R.M. (2014). Social Media Effects on Young Women’s Body Image Concerns: Theoretical Perspectives and an Agenda for Research. Sex Roles.

[35] Wall Street Journal (2021). Investigation: How TikTok’s algorithm figures out your deepest desires. The Wall Street Journal.

[36] Wells G., Horwitz J., Seetharaman D. (2021). Facebook knows Instagram is toxic for many teen girls, company documents show. The Wall Street Journal.

[37] Mac R., Kang C. (2021). Whistle-blower says Facebook ‘chooses profits over safety’. The New York Times.

[38] Griffiths M.D., Kuss D. (2017). Adolescent social media addiction (revisited). Educ. Health.

[39] Smith B. (2021). How TikTok reads your mind. The New York Times.

[40] Kleemans M., Daalmans S., Carlier M., Jansen A. (2022). Picture perfect: The direct and mediated effect of manipulated Instagram photos on body image dissatisfaction. Body Image.

[41] Walker C.E., Krumhuber E.G., Dayan S., Furnham A. (2021). Effects of social media use on desire for cosmetic surgery among young women. Curr. Psychol..

[42] (2021). Surgical Procedure Demand Skyrockets & Video Conferencing Takes “Selfie Awareness” to the Next Level. https://www.aafprs.org/Media/Press_Releases/PageTemplates/New%20Survey%20Results%20Announced%20Feb.%201,%202021.aspx.

[43] Wang Y., Fardouly J., Vartanian L.R., Lei L. (2019). Selfie-viewing and facial dissatisfaction among Chinese adolescents: A moderated mediation model of general attractiveness internalization and body appreciation. Body Image.

[44] Fardouly J., Rapee R.M. (2019). The impact of no-makeup selfies on young women’s body image. Body Image.

[45] Mabe K.J., Forney P.K., Keel P.K. (2014). Do you “like” my photo? Facebook use maintains eating disorder risk. Int. J. Eat. Disord..

[46] Avalos L., Tylka T.L., Wood-Barcalow N. (2005). The Body Appreciation Scale: Development and psychometric evaluation. Body Image.

[47] Wood-Barcalow N.L., Tylka T.L., Augustus-Horvath C.L. (2010). “But I like my body”: Positive body image characteristics and a holistic model for young-adult women. Body Image.

[48] Frisén A., Holmqvist K. (2010). What characterizes early adolescents with a positive body image? A qualitative investigation of Swedish girls and boys. Body Image.

[49] Holmqvist K., Frisén A. (2012). “I bet they aren’t that perfect in reality”: Appearance ideals viewed from the perspective of adolescents with a positive body image. Body Image.

[50] Tylka T.L., Wood-Barcalow N.L. (2015). The Body Appreciation Scale-2: Item refinement and psychometric evaluation. Body Image.

[51] Tylka T.L., Wood-Barcalow N.L. (2015). A positive complement. Body Image.

[52] Rodgers R.F., Wertheim E.H., Paxton S.J., Tylka T.L., Harriger J.A. (2022). Bopo: Enhancing body image through body positive social media-evidence to date and research directions. Body Image.

[53] Griffin M., Bailey K.A., Lopez K.J. (2022). #BodyPositive? A critical exploration of the body positive movement within physical cultures taking an intersectionality approach. Front. Sports Act. Living.

[54] Afful A.A., Ricciardelli R. (2015). Shaping the online fat acceptance movement: Talking about body image and beauty standards. J. Gend. Stud..

[55] Goodman E. (2023). When Did Body Positivity Get Toxic?. https://renfrewcenter.com/in-the-media-when-did-body-positivity-get-toxic/.

[56] Wood-Barcalow N.L., Alleva J.M., Tylka T.L. (2024). Revisiting positive body image to demonstrate how body neutrality is not new. Body Image.

[57] Smith J.A., Flowers P., Larkin M. (2009). Interpretative Phenomenological Analysis: Theory, Method and Research.

[58] Smith J.A., Osborn M., Smith J. (2015). Interpretative phenomenological analysis. Qualitative Psychology: A Practical Guide to Research Methods.

[59] Pietkiewicz I., Smith J.A. (2014). A practical guide to using interpretative phenomenological analysis in qualitative research psychology. Psychol. J..

[60] Shaw R. (2010). Embedding reflexivity within experiential qualitative psychology. Qual. Res. Psychol..

[61] Newton B.J., Rothlingova Z., Gutteridge R., LeMarchand K., Raphael J.H. (2012). No room for reflexivity? Critical reflections following a systematic review of qualitative research. J. Health Psychol..

[62] Willson E., Kerr G. (2022). Body shaming as a form of emotional abuse in sport. Int. J. Sport Exerc. Psychol..

[63] Bartky S. (1990). Femininity & Domination: Studies in the Phenomenology of Oppression.

[64] Holland G., Tiggemann M. (2016). A systematic review of the impact of thin ideal media exposure on body image in women. Clin. Psychol. Rev..

[65] Kellie D.J., Blake K.R., Brooks R.C. (2019). What drives female objectification? An investigation of appearance-based interpersonal perceptions and the objectification of women. PLoS ONE.

[66] Bonell S., Barlow F.K., Griffiths S. (2021). The cosmetic surgery paradox: Toward a contemporary understanding of cosmetic surgery popularisation and attitudes. Body Image.

[67] Kuczynski A. (2006). Beauty Junkies: Inside Our $15 Billion Obsession with Cosmetic Surgery.

[68] Aniulis E., Sharp G., Thomas N.A. (2021). The ever-changing ideal: The body you want depends on who else you’re looking at. Body Image.

[69] Tragantzopoulou P. (2021). The long-lasting cycle of transformations in eating habits and the emergence of Orthorexia Nervosa: Covid-19 implications and future challenges. Sention J..

[70] Tragantzopoulou P., Fixsen A., Ridge D., Cheshire A. (2024). “You are not alone, we’ve got you”: Power plays, devotion, and punishment on healthy eating and pro-eating disorder websites. Qual. Health Res..

[71] Jong S.T., Drummond M.J.N. (2016). Exploring online fitness culture and young females. Leis. Stud..

[72] Boepple L., Ata R.N., Rum R., Thompson J.K. (2016). Strong is the new skinny: A content analysis of fitspiration websites. Body Image.

[73] Tiggemann M., Zaccardo M. (2018). ’Strong is the new skinny’: A content analysis of #fitspiration images on Instagram. J. Health Psychol..

[74] Tragantzopoulou P., Giannouli V. (2020). Eating disorders and body image disturbance among males and females: From the perspective of six therapists with different therapeutic orientations. Anthropol. Res. Stud..

[75] Tragantzopoulou P., Giannouli V. (2023). “You feel that you are stepping into a different world”: Vulnerability and biases in the treatment of anorexia nervosa. Eur. J. Psychother. Counsel..

[76] Tragantzopoulou P., Mouratidis C., Paitaridou K., Giannouli V. (2024). The Battle Within: A Qualitative Meta-Synthesis of the Experience of the Eating Disorder Voice. Healthcare.

[77] Tragantzopoulou P., Giannouli V. (2024). Unveiling Anxiety Factors in Orthorexia Nervosa: A Qualitative Exploration of Fears and Coping Strategies. Healthcare.

[78] Halliwell E., Dittmar H. (2003). A qualitative investigation of women’s and men’s body image concerns and their attitudes toward aging. Sex Roles.

